# Cisplatin-Resistant Urothelial Bladder Cancer Cells Undergo Metabolic Reprogramming beyond the Warburg Effect

**DOI:** 10.3390/cancers16071418

**Published:** 2024-04-05

**Authors:** Julieta Afonso, Catarina Barbosa-Matos, Ricardo Silvestre, Joana Pereira-Vieira, Samuel Martins Gonçalves, Camille Mendes-Alves, Pier Parpot, Joana Pinto, Ângela Carapito, Paula Guedes de Pinho, Lúcio Santos, Adhemar Longatto-Filho, Fátima Baltazar

**Affiliations:** 1 Life and Health Sciences Research Institute (ICVS), Campus de Gualtar, University of Minho, 4710-057 Braga, Portugal; id8801@alunos.uminho.pt (C.B.-M.); ricardosilvestre@med.uminho.pt (R.S.); id8183@alunos.uminho.pt (J.P.-V.); samuelgoncalves@med.uminho.pt (S.M.G.); longatto@med.uminho.pt (A.L.-F.); fbaltazar@med.uminho.pt (F.B.); 2 ICVS/3B’s—PT Government Associate Laboratory, 4710-057 Braga, Portugal; 3 CQUM, Centre of Chemistry, Chemistry Department, Campus de Gualtar, University of Minho, 4710-057 Braga, Portugal; camille.MENDES-ALVES@etu.univ-amu.fr (C.M.-A.); parpot@quimica.uminho.pt (P.P.); 4 CEB—Centre of Biological Engineering, Campus de Gualtar, University of Minho, 4710-057 Braga, Portugal; 5 Associate Laboratory i4HB—Institute for Health and Bioeconomy, University of Porto, 4050-313 Porto, Portugal; jipinto@ff.up.pt (J.P.); up201804198@up.pt (Â.C.); pguedes@ff.up.pt (P.G.d.P.); 6 UCIBIO—Applied Molecular Biosciences Unit, Laboratory of Toxicology, Faculty of Pharmacy, University of Porto, 4050-313 Porto, Portugal; 7 Experimental Pathology and Therapeutics Group, Research Center of the Portuguese Institute of Oncology (CI-IPOP), 4200-072 Porto, Portugal; llarasantos@gmail.com; 8 Porto Comprehensive Cancer Center (P.CCC), 4200-072 Porto, Portugal; 9 Laboratory of Medical Investigation (LIM14), Faculty of Medicine, São Paulo State University, São Paulo 01049-010, Brazil; 10 Molecular Oncology Research Center, Barretos Cancer Hospital, São Paulo 14784-400, Brazil

**Keywords:** urothelial bladder cancer, cisplatin resistance, Warburg effect, glucose, lactate, lipid metabolism, glutamate

## Abstract

**Simple Summary:**

Cisplatin-based resistance is an old concern for advanced urothelial bladder cancer (UBC) patients. This work aimed to study the metabolic profiles of three pairs of cisplatin-sensitive (CS) and cisplatin-resistant (CR) UBC cell lines. A tendency was observed towards an intensified glycolytic metabolism in two of the CR cell lines, especially in the CR cells showing higher levels of cisplatin resistance. However, in this cell line, a shift towards alternative metabolic routes involving lactate uptake, lipid biosynthesis and glutamate metabolism occurred with time. The remaining cell line seemed to engage in a metabolic reprogramming, recovering the preference for oxygen consumption. Thus, CR UBC cells displayed deep metabolic alterations that likely depended on their molecular backgrounds.

**Abstract:**

Advanced urothelial bladder cancer (UBC) patients are tagged by a dismal prognosis and high mortality rates, mostly due to their poor response to standard-of-care platinum-based therapy. Mediators of chemoresistance are not fully elucidated. This work aimed to study the metabolic profile of advanced UBC, in the context of cisplatin resistance. Three isogenic pairs of parental cell lines (T24, HT1376 and KU1919) and the matching cisplatin-resistant (R) sublines were used. A set of functional assays was used to perform a metabolic screening on the cells. In comparison to the parental sublines, a tendency was observed towards an exacerbated glycolytic metabolism in the cisplatin-resistant T24 and HT1376 cells; this glycolytic phenotype was particularly evident for the HT1376/HT1376R pair, for which the cisplatin resistance ratio was higher. HT1376R cells showed decreased basal respiration and oxygen consumption associated with ATP production; in accordance, the extracellular acidification rate was also higher in the resistant subline. Glycolytic rate assay confirmed that these cells presented higher basal glycolysis, with an increase in proton efflux. While the results of real-time metabolomics seem to substantiate the manifestation of the Warburg phenotype in HT1376R cells, a shift towards distinct metabolic pathways involving lactate uptake, lipid biosynthesis and glutamate metabolism occurred with time. On the other hand, KU1919R cells seem to engage in a metabolic rewiring, recovering their preference for oxidative phosphorylation. In conclusion, cisplatin-resistant UBC cells seem to display deep metabolic alterations surpassing the Warburg effect, which likely depend on the molecular signature of each cell line.

## 1. Introduction

Bladder cancer ranks as one of the most frequently diagnosed malignancies worldwide, particularly in the male gender, where it holds the 6th and 9th positions in terms of incidence and mortality rates, respectively [1]. Urothelial bladder carcinoma (UBC) is the main histological variant [2]. Occupational exposure to aromatic amines and lifelong smoking habits are the most important risk factors for the development of this disease and, for that reason, its incidence increases with age [3]. Divergent histopathological and behavioral profiles separate non-muscle invasive (NMI, about 70% of total) from muscle-invasive (MI, about 30% of total) tumors [2]. Importantly, UBC represents a remarkable economic impact in health care systems [4], mostly due to the frequent recurrences and stage progression of NMI tumors and the resistance to standard-of-care cisplatin-based therapy of patients portending MI disease [2]. In this latter group, the prognosis is aggravated by comorbidities and frailty typical of the elderly population, with the median overall survival being approximately 15 months for metastatic UBC patients [5]. PD-1-/PD-L1-directed immunotherapy and other targeted therapies have brought fresh air to the therapeutic armamentarium for MI and metastatic bladder cancer [6], but cisplatin-based combination chemotherapy remains the first-line treatment option for UBC-eligible patients [7]. In an era where the UBC therapeutic paradigm is evolving, robust biomarkers that can predict a response to therapy and anticipate resistance events are urgently needed.

Cisplatin is a platinum-based agent widely used for the treatment of numerous malignancies, namely bladder cancer, but also head and neck, ovarian, cervical and lung cancers, among others. It exerts its anticancer activity by inducing multiple physiological alterations. Its primary mechanism of action involves the generation of DNA adducts on nuclear or mitochondrial DNA, which blocks DNA replication, mRNA production and protein translation, and activates alternative transduction pathways, ultimately leading to apoptosis or necrosis [8]. Although cisplatin is one of the most potent anticancer drugs, its usage is often limited by severe side toxicities and drug resistance events. Dissecting these events and their targets is essential to tailoring the treatment scheme based on individual resistance profiles. Resistance mechanisms in bladder cancer are shared by other malignancies and include increased DNA repair and drug efflux, modifications in cell cycle and cell death signaling pathways, dysregulation in protein glycosylation and clearance, ferroptosis inhibition, epigenetic events, and metabolic reprogramming [9,10]. Regarding this last aspect, aberrant glucose metabolism and oxidative phosphorylation are hallmarks of cancer cells [11] and appear to contribute to cisplatin resistance [12,13]. Thus, these hallmarks might harbor potential predictive biomarkers of response and new therapeutic targets.

To meet the heightened bioenergetic, biosynthetic and redox demands necessary to support tumor growth, cancer cells adopt the Warburg phenotype by upregulating glycolytic machinery and producing massive amounts of lactate, even under high oxygen tension [14,15]. Bladder cancer, similarly to other malignancies, exhibits this glycolytic phenotype (reviewed in [16,17,18]). Several studies reported significant associations between aggressive tumor features and poor prognosis, and the upregulation of glucose transporters [19], glycolytic enzymes and their downstream players [20,21,22,23,24], pH regulators [25], and lactate transporters [26,27]. Genetic and/or pharmacological inhibition of these molecules suppressed aerobic glycolysis, cell proliferation, migration and invasion in vitro and tumor growth in vivo [20,23,24,28,29,30,31,32]. Interestingly, a few authors have also described that glycolytic bladder cancer cells are prone to developing cisplatin resistance. Li et al. showed that microRNA-218 targets GLUT1, inhibiting glucose uptake while promoting the sensitivity of UBC cell lines to cisplatin; conversely, chemoresistance was restored upon GLUT1 upregulation [33]. UBC chemosensitivity to cisplatin was increased upon inhibition of the catalysts hexokinase 2 (HK2) [24] and pyruvate kinase M2 (PKM2) [30]. Similarly, downregulation of pyruvate dehydrogenase kinase 4 (PDK4) reverted the inhibition of the pyruvate dehydrogenase complex and reduced cell proliferation and tumor volume, potentiating cisplatin effects both in vitro and in vivo [23]. Inhibition of CD147, an obligate chaperone for the function of monocarboxylate transporters (MCTs) 1 and 4, which facilitate lactate release from highly glycolytic cells, restored cisplatin sensitivity in vitro. We previously reported a partnership between MCT1 and CD147 in promoting worse survival rates in UBC patients treated with cisplatin-based regimens [26] and established a connection between a profile of metabolic symbiosis among UBC cells and stromal cells with cisplatin resistance [27]. The evidence shown above of the association between a glycolytic phenotype and cisplatin resistance in UBC is based on the study of individual glycolytic biomarkers, and exploitation of the glycolytic metabolism program in a suitable model of cisplatin resistance is missing. In this work, using a panel of isogenic pairs of cisplatin-sensitive versus cisplatin-resistant MI-UBC cells, we aimed to perform a comprehensive characterization of the metabolic profile of these cells in the context of cisplatin resistance, with an emphasis on the glycolytic metabolism and the Warburg effect.

## 2. Materials and Methods

### 2.1. Cell Lines and General Cell Culture Procedures

In the present study, three isogenic pairs of muscle-invasive UBC cell lines were used. T24, HT1376 and KU1919 parental cell lines were obtained from the American Type Culture Collection (ATCC); their cisplatin-resistant (R) counterparts (T24^r^CDDP^1000^, HT1376^r^CDDP^1000^ and KU1919^r^CDDP^1000^) are part of the Resistant Cancer Cell Line (RCCL) collection (www.wass-michaelislab.org/rccl.php (accessed on 12 January 2023)) [34] and were obtained through an intermittent stepwise selection protocol, as described previously [35]. Short-tandem repeat (STR) profiling was used for cell line authentication. The cell lines were cultured in Iscove’s Modified Dulbecco’s Medium (IMDM; Sigma-Aldrich^®^, St. Louis, MO, USA), supplemented with 10% fetal bovine serum (FBS; Sigma-Aldrich^®^, St. Louis, MO, USA) and 1% penicillin/streptomycin solution (GRiSP; Porto, Portugal) (unless otherwise specified), being maintained in a humidified atmosphere at 37 °C and 5% CO_2_ and regularly subcultured. Cisplatin was added to the culture medium when subculturing the cisplatin-resistant sublines (1 μg/mL—cisplatin concentration to which the cells were adapted) to maintain the selection pressure; the drug was removed three passages prior to the experiments, so that its possible acute effects could be avoided. Stock solutions of 1 mg/mL cisplatin (CDDP, cis-diamminedichloroplatinum(II) in 10% NaCl were donated by the Pharmaceutical Services of the Portuguese Institute of Oncology (IPO), Porto, Portugal (leftovers from oncological treatment).

### 2.2. Cell Viability Assay

To confirm the cisplatin-resistant phenotype of the R cells and determine the cisplatin resistance ratio of each pair, we assessed the chemosensitivity of the isogenic pairs of UBC cell lines to CDDP. For this, cells were seeded in triplicate into 48-well plates at a density of 1.2 × 10^4^ (T24 and KU1919 pairs) or 1.4 × 10^4^ (HT1376) cells/well and incubated for 48 h. The medium was then removed and replaced with fresh medium (FBS-free) containing ascending concentrations of CDDP (0–10 μg/mL). Following 72 h incubation, the effect of the CDDP treatment on cellular viability was determined by a sulforhodamine B assay (SRB, TOX-6; Sigma-Aldrich^®^, St. Louis, MO, USA), according to the manufacturer’s instructions. A spectrophotometer (Varioskan^®^ Flash; Thermo Fisher Scientific, Waltham, MA, USA) was used to measure absorbance, at a wavelength of 490 nm. The results are expressed as the mean percentages ± SDs of viable cells relatively to control conditions (considered as 100% viability). IC_50_ values were determined by nonlinear regression analysis, using GraphPad Prism 8.4.2 software.

### 2.3. Protein Extraction and Western Blotting

UBC cells grown to 90% confluence in 6-well plates were washed in cold PBS, scrapped and homogenized in lysis buffer for 10 min on ice and centrifuged at 13,000 rpm, at 4 °C for 15 min. The Bradford assay (Sigma-Aldrich^®^, St. Louis, MO, USA) was used to quantify protein content. Equal amounts (20 μg) of total protein were separated at 100 V for 90 min on a 12% polyacrylamide gel by SDS-PAGE and then transferred to nitrocellulose membranes at 25 V for 30 min in a Trans-Blot^®^ Turbo^TM^ Transfer System (Bio-Rad Laboratories, Hercules, CA, USA). After a blocking step (5% milk in 1 × TBS for 60 min), membranes were incubated with the primary antibodies (including loading controls) overnight (ON) at 4 °C, and with appropriate secondary antibodies at room temperature (RT) during 1 h (Table S1). Immunoreactive bands were visualized with enhanced chemiluminescence (SuperSignal West Femto kit; Pierce^TM^, Thermo Fisher Scientific, Waltham, MA, USA) on a ChemiDoc^TM^ XRS+ system (Bio-Rad Laboratories, Hercules, CA, USA).

### 2.4. Immunofluorescence

UBC cells (4.5 × 10^4^ cells/well) were seeded in 12-well plates previously coated with round coverslips, and incubated during 24 h. Cells were then fixed and permeabilized with cold methanol for 20 min at −20 °C. After a blocking step in 5% BSA during 30 min, cells were incubated with the primary (4 °C ON) and fluorescence-conjugated secondary antibodies (1 h RT; Table S1). Cells were mounted in Vectashield^®^ Mounting Media with 4′,6-diamidino-2-phenylindole (DAPI) (Vector Laboratories, Inc., Newark, CA, USA), and images were captured by a fluorescence microscope (BX61; Olympus^®^, Tokyo, Japan).

### 2.5. Glucose Uptake Analysis by Flow Cytometry

UBC cells were seeded in 6-well plates (4 × 10^5^ cells/well) in glucose-free Dulbecco’s Modified Eagle Medium (DMEM) during 16 h; then, cells were incubated with 10 μM (T24 and KU1919 pairs) or 100 μM (HT1376 pair) of 2-deoxy-2-((7-nitro-2,1,3-benzoxadiazol-4-yl)amino)-D-glucose (2-NBDG; Molecular Probes^®^, Eugene, OR, USA), during 2 h (T24 and KU1919 pairs) or 5 h (HT1376 pair), at 37 °C and protected from light. After incubation, cells were washed with 1 × PBS, and data were acquired with a flow cytometer (LSRII; BD Biosciences, San Jose, CA, USA) and analyzed with FlowJo v10 analytical software (TreeStar, Inc., San Francisco, CA, USA). Mean fluorescence intensities ± SEMs are reported.

### 2.6. Colorimetric Analysis of Extracellular Glucose

UBC cells were seeded in triplicate in 48-well plates (T24 and KU1919 pairs: 6 × 10^4^ cells/well; HT1376 pair: 9 × 10^4^ cells/well) and allowed to adhere overnight. Spent culture medium was replaced by fresh medium, and after 24 h, extracellular glucose content was analyzed in the cell culture medium using a commercial enzymatic colorimetric kit (Spinreact, Girona, Spain). Absorbance was measured spectrophotometrically at a wavelength of 490 nm (Varioskan^®^ Flash; Thermo Fisher Scientific, Waltham, MA, USA). Blank absorbance (fresh culture medium only) was measured and subtracted from the test measurements. The results were normalized for this time point by the total protein amount (expressed as the total biomass), assessed using the SRB assay (TOX-6; Sigma-Aldrich^®^, St. Louis, MO, USA).

### 2.7. ATP Quantification by Bioluminescence

UBC cells were plated in triplicate in 6-well plates (1 × 10^6^ cells/well) and allowed to adhere for 24 h. Cell lysates were obtained to quantitatively determine ATP content by bioluminescence, using recombinant firefly luciferase and D-luciferin substrate, according to the instructions from the manufacturer (Molecular Probes^TM^, Eugene, OR, USA). A hybrid multi-mode microplate reader with luminescence detection mode (Synergy^TM^ H1; BioTek, Winooski, VT, USA) was used to measure luminescence. Protein content determined by the Bradford assay (Sigma-Aldrich^®^, St. Louis, MO, USA) was used to normalize the results.

### 2.8. Analysis of Mitochondrial Activity by Flow Cytometry

UBC cells were seeded in 6-well plates (4 × 10^5^ cells/well) and allowed to adhere during 24 h, followed by a 2 h incubation at 37 °C with 200 nM of the molecular probes MitoTracker Red (MR) and MitoTracker Green (MG), both from Molecular Probes^®^, Eugene, OR, USA, protected from light. Cells were then washed with 1 × PBS, and data were acquired with a flow cytometer (LSRII; BD Biosciences, San Jose, CA, USA) and analyzed with the FlowJo v10 analytical software (TreeStar, Inc., San Francisco, CA, USA). MR and MG were used to measure mitochondrial polarization and mitochondrial mass, respectively. Mitochondrial activity was given by the mitochondrial polarization/mitochondrial mass ratio.

### 2.9. Fluorometric Analysis of Reactive Oxygen Species (ROS) Production

UBC cells were seeded in 48-well plates (T24 pair: 8 × 10^4^ cells/well; HT1376 pair: 9 × 10^4^ cells/well; KU1919 pair: 6 × 10^4^ cells/well) and allowed to adhere during 24 h, followed by a 30 min incubation at 37 °C with 5 μM of 2′,7’–dichlorofluorescin diacetate (CM-H2DCFDA; Thermo Fisher Scientific, Waltham, MA, USA) in 1 × PBS, protected from light. The results were obtained by live cell imaging fluorescence, in an IncuCyte^®^ Live-Cell Analysis System (Sartorius, Göttingen, Germany). Images were acquired with a 20× objective. Phase images were acquired for every condition. For green fluorescence imaging, the acquisition time was 300 ms. Analysis was performed with the IncuCyte^®^ Cell-by-Cell software module v2022A (Sartorius, Göttingen, Germany).

### 2.10. Colorimetric Analysis of Glutathione Content

UBC cells were plated in T25 flasks and allowed to grow to confluence. Cells were then harvested by trypsinization, and part of the volume was used for protein quantification; the remaining (at least 1 × 10^6^ cells) was pelleted by centrifugation and resuspended in a 5% sulfosalicylic acid solution, for deproteinization. A glutathione colorimetric detection kit was then used to measure glutathione (GSH) and oxidized glutathione (GSSG) content, according to the manufacturer’s instructions (Invitrogen^TM^, Thermo Fisher Scientific, Waltham, MA, USA). A 2-vinylpyridine solution was used to block free GSH or other thiols present in the samples, to determine GSSG content. A spectrophotometer (Varioskan^®^ Flash; Thermo Fisher Scientific, Waltham, MA, USA) was used to measure absorbance at 405 nm. Protein content, determined by the Bradford assay (Sigma-Aldrich^®^, St. Louis, MO, USA), was used for normalization of the results.

### 2.11. Analysis of Neutral Lipid Content and Palmitate Uptake by Flow Cytometry

UBC cells were seeded in 24-well plates (T24 and KU1919 pairs: 1.2 × 10^5^ cells/well; HT1376 pair: 1.5 × 10^5^ cells/well) and allowed to adhere overnight. Spent culture medium was replaced by fresh medium, and after 24 h, cells were harvested by trypsinization, followed by incubation during 30 min in the dark at 37 °C with 3 μg/mL BODIPY™ 493/503 (Thermo Fisher Scientific, Waltham, MA, USA) or 1 μM BODIPY™ FL C_16_ (Thermo Fisher Scientific, Waltham, MA, USA), for the analysis of intracellular neutral lipid content or palmitate uptake, respectively, by flow cytometry, as previously described [36].

### 2.12. Seahorse Analysis of Mitochondrial Respiration

The cellular oxygen consumption rate (OCR) and extracellular acidification rate (ECAR) were measured in a Seahorse XFe96 Extracellular Flux Analyzer (Agilent Technologies, Inc., Santa Clara, CA, USA) using the XF Cell Mito Stress Test and XF Glycolytic Rate Assay kits (XFp; Seahorse Bioscience, Billerica, MA, USA). HT1376-paired cells were plated on XFp 96-well microplates (5 × 10^4^ cells/well) and allowed to adhere overnight. The sensor cartridges for the XFp analyzer were hydrated in a 37 °C non-CO_2_ incubator a day before the experiment. For the Mito Stress Test, the injection port A on the sensor cartridge was loaded with 3.0 μM oligomycin, while 0.25 μM FCCP was loaded on port B and 1.0 μM rotenone/antimycin A wasere loaded on port C. For the Glycolytic Rate Assay, the conditions used were the following: injection of 1.0 μM rotenone/antimycin A on port A and 50.0 mM 2-deoxy-D-glucose on port B. During sensor calibration, cells were incubated in a 37 °C non-CO_2_ incubator in 175 μL of the Mito Stress medium (25 mM glucose, 1 mM pyruvate and 4 mM L-glutamine; pH 7.4) or Glycolytic Rate Assay medium (10 mM glucose, 1 mM pyruvate and 2 mM L-glutamine; pH 7.4). The plate was immediately placed onto the calibrated XFp Extracellular Flux Analyzer for the respective tests. The results were normalized for the total proteins/well (expressed as the total biomass), assessed using the SRB assay (TOX-6; Sigma-Aldrich^®^, St. Louis, MO, USA).

### 2.13. Chromatographic Analysis of Extracellular Glucose, Pyruvate and Lactate

UBC cells from the HT1376 cell line pair were seeded in triplicate in 12-well plates (2.2 × 10^5^ cells/well) and allowed to adhere overnight. Spent culture medium was replaced by fresh medium, and after 24 h, the medium of each well was transferred to Eppendorf tubes. Following deproteinization (perchloric acid precipitation) and filtration of the cell culture medium samples, high-performance liquid chromatography–mass spectrometry (HPLC–MS) was used to detect basal levels of glucose, pyruvate and lactate contents. For this, a Thermo Finnigan Surveyor liquid chromatograph coupled to an LXQ linear ion trap mass spectrometer (Finnigan, San Jose, CA, USA), equipped with an electrospray ionization (ESI) source, was used; Xcalibur Quant software (version 2.7) was used for instrument control and data acquisition. The HPLC–MS system contained a photodiode array detector (PDA), an autosampler plus system and an E2M30 pump (Edwards, West Sussex, UK). The product partition was carried out on a Synergi 4u Hydro-RP 80A, 150 × 4.6 mm analytical column (Phenomenex, Torrance, CA, USA). The mobile phase was composed of (A) milli-Q water with 0.1% (*v*/*v*) formic acid and (B) acetonitrile. The elution was performed at a flow rate of 0.3 mL/min, in gradient mode (initial: 80% A and 20% B; 15 min: 10% A and 90% B; 25 min: 80% A and 20% B), with these last conditions being maintained until the end of the analysis (30 min). A volume of 25 μL per sample was injected. The sheath gas flow rate, auxiliary gas flow rate, sweep gas flow rate, tube lens, capillary Voltage, capillary temperature and spray voltage (source-dependent parameters) were optimized to 50, 10, 10, 115 V, 46 V, 275 °C and 5000 V, respectively. A quantitative analysis was carried out by HPLC–UV/RID, using an isocratic pump (Jasco PU-980; JASCO Corporation, Tokyo, Japan) and double online detection including a UV–Vis detector (wavelength = 210 nm, Jasco UV-975; JASCO Corporation, Tokyo, Japan) and refractometer (Shimadzu-RID 6A; Shimadzu Corporation, Kyoto, Japan). The product partition was performed on an ion exchange column, Rezex Organic Acid H^+^ from Phenomenex (Torrance, CA, USA). The eluent was H_2_SO_4_ (0.2 mL/L) in this case. The results were normalized for the total proteins/well (expressed as the total biomass), assessed using the SRB assay (TOX-6; Sigma-Aldrich^®^, St. Louis, MO, USA).

### 2.14. Proton Nuclear Magnetic Resonance (^1^H NMR)-Based Metabolomics for Intracellular Metabolome Analysis

UBC cells from the HT1376 cell line pair were seeded in 60 mm cell culture dishes (1.5 × 10^6^ cells/dish) and allowed to adhere overnight. Spent culture medium was replaced by fresh medium and the cells were further incubated for 24 h; the medium was then discarded and the cells were processed for intracellular metabolome analysis according to a modified dual-phase extraction method [37,38]. Briefly, after three washing steps with NaCl 0.9%, adherent cells were scrapped/quenched with cold methanol (800 μL) and transferred to Eppendorf tubes; the cells were then sonicated three times for 30 s at 23 kHz and a 10 μm amplitude using an exponential probe, with 30 s of rest between sonication. This step was followed by vortexing for 2 min for further cell breakage. Afterwards, chloroform (320 μL, added twice) and water (296 μL) were added in between 2 min vortexing steps, followed by 10 min on ice and centrifugation (2000 × *g* for 15 min plus 10,000 × g for 2 min, at 4 °C). The polar metabolite-containing upper phase was then collected, lyophilized and stored at −80 °C until all independent replicates were obtained. For ^1^H NMR analysis, 650 μL of phosphate buffer 100 mM pH 7.4 with 0.05 mM TSP (100 % deuterium oxide (D_2_O)) was added to each lyophilized extract, followed by centrifugation at 10,410× *g* for 5 min at 4 °C. Analysis was performed on a Bruker Avance III HD 600 MHz spectrometer (Bruker BioSpin; Rheinstetten, Germany) equipped with a cryoprobe at 298 K, with recording of ^1^H standard 1D spectra (*noesypr1d*) (relaxation delay: 4 s; mixing time: 100 ms; transients: 256; complex data points: 64 k; spectral width: 10,080.646 Hz; acquisition time: 3.25 s); these spectra were processed with a 1.0 Hz exponential line-broadening function, manually phased and baseline corrected, and chemical shifts referenced to lactate at δ = 1.32 ppm. Samples were compared for their ^1^H resonances with the ^1^H NMR spectra of standard compounds (Biological Magnetic Resonance Bank [39] and Chenomx NMR suite 8.4 software; Chenomx Inc., Edmonton, AB, Canada). Spectral data pre-processing was performed with the NMRProcFlow 1.4 online tool [40]. The spectral regions of residual water (5.20–4.67 ppm) and methanol (3.36–3.34) were excluded from the whole spectral region between 9.35 and 0.70 ppm. Finally, the spectra of the intracellular extracts were aligned according to the parametric time warping method [41], followed by bucketing with a uniform spectral width (0.001 ppm, signal to noise ratio = 3) and normalization by the total area.

### 2.15. Statistical Analysis

All results were statistically analyzed using the GraphPad Prism 8.4.2 software (San Diego, CA, USA.), with the Student’s *t*-test or two-way ANOVA, considering significant *p*-values lower than 0.05 (* *p* < 0.05; ** *p* < 0.01; *** *p* < 0.005; **** *p* < 0.001). At least three independent experiments were performed in each assay.

Regarding the analysis of the ^1^H NMR data, the final matrices were scaled to unit variance, and multivariate analysis methods (principal component analysis (PCA) and partial least squares discriminant analysis (PLS-DA)) were considered, using SIMCA 13 (Umetrics, Umea, Sweden) software. The performance of the PLS-DA models was confirmed by a default sevenfold internal cross-validation, which retrieved the number of latent variables (LVs), fraction of X-explained variance (R^2^X), fraction of Y-explained variance (R^2^Y) and fraction of Y variation predicted by the X model or predictive ability (Q^2^). The PLS-DA loading plots were created in R 4.0.3 software [42], with the ggplot2 package [43]. Appropriate resonances (variable importance for the projection (VIP) > 1.0) were evaluated using univariate statistical methods (*t*-test and Mann–Whitney test). A *p*-value less than 0.05 was applied to establish statistical significance.

## 3. Results

### 3.1. Effect of Cisplatin on Cell Viability

**Figure 1:**
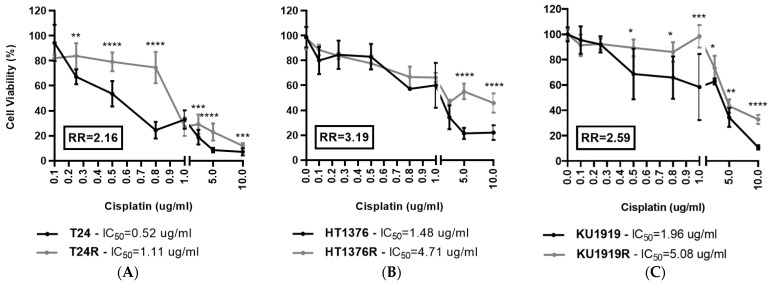
Viability of isogenic pairs of urothelial bladder cancer cell lines ((**A**), T24 pair; (**B**), HT1376 pair; (**C**), KU1919 pair) exposed to increasing concentrations of cisplatin during 72 h. * *p* < 0.05, ** *p* < 0.01, *** *p* < 0.005, **** *p* < 0.001, parental cells versus cisplatin-resistant cells at each indicated cisplatin concentration. [IC_50_, half-maximal inhibitory concentration; R, cisplatin-resistant cell line; RR, resistance ratio (IC_50_ cisplatin-resistant cell line/IC_50_ parental cell line)].

In order to characterize the chemosensitivity of three isogenic pairs of muscle-invasive UBC cell lines to cisplatin, IC_50_ values were estimated after 72 h of exposure to the drug (Figure 1A–C). We confirmed the resistance phenotype of the cisplatin-resistant cells as evidenced by the significant differences in cell viability and their higher IC_50_ values compared to their parental counterparts. The T24 (Figure 1A) and HT1376 (Figure 1B) pairs showed the lowest and highest resistance ratios, respectively. This refers to the ratio of IC_50_ values between the cisplatin-resistant and the parental cell lines. Interestingly, the cell viability of HT1376R cells was significantly higher than HT1376 only at the highest cisplatin concentrations (Figure 1B).

### 3.2. Immunoexpression of Glycometabolism and Cell Death-Related Biomarkers

**Figure 2:**
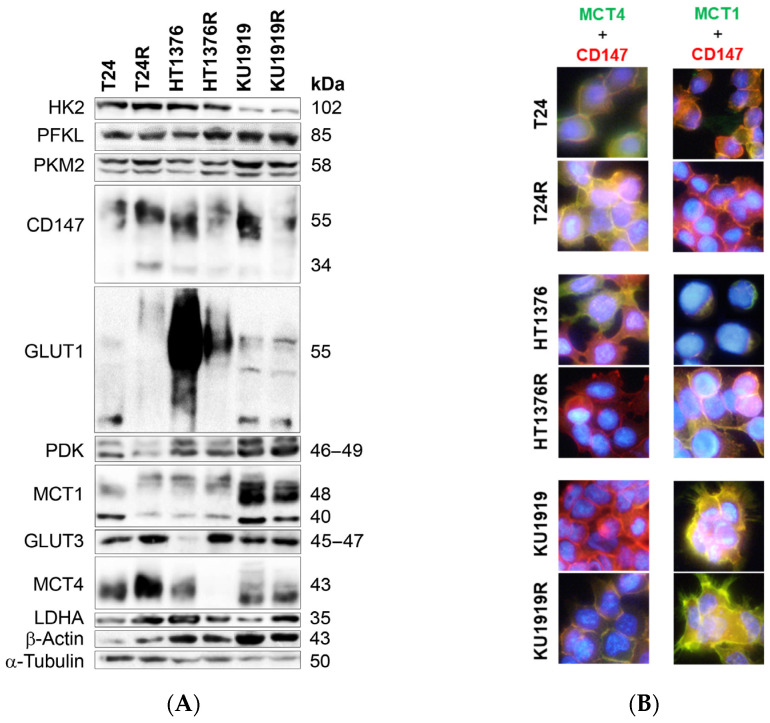
Western blot (**A**) and immunofluorescence (**B**) of baseline levels of glycometabolism biomarkers in isogenic pairs of urothelial bladder cancer cell lines. The Western blot results (**A**) are representative of similar blots from three independent cell lysates (quantification of the results shown in Figure S2). In (**B**), co-localization of MCT4 and MCT1 (green; respectively) with CD147 (red) is shown in yellow. Cell nuclei were counterstained with DAPI (blue), and pictures were obtained at 400× amplification (scale bar).

Western blotting of glycometabolism biomarkers (Figure 2A) revealed no apparent differences between the parental and cisplatin-resistant counterparts of all the isogenic pairs of cell lines, with the exception of the HT1376 pair. HT1376R cells exhibited a lower expression of MCT4 and GLUT1 than HT1376 and a higher expression of GLUT3. The results regarding MCT4 expression were validated through immunofluorescence (MCT4, Figure 2B). As expected, MCT1 and MCT4 (Figure 2B) co-localized with CD147 in all of the cell lines, both at the plasma membrane and the cytoplasm. Regarding the immunoexpression of cell death-related biomarkers (Figure S1), no differences were observed among the isogeneic pairs.

### 3.3. Glycolytic Metabolism and Mitochondrial Function

**Figure 3:**
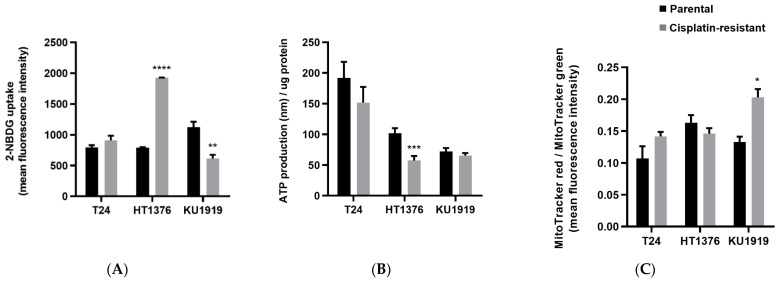
Glycolytic metabolism and mitochondrial function of isogenic pairs of urothelial bladder cancer cell lines. The 2-NBDG uptake (**A**) was assessed after 2 h (T24 and KU1919 pairs)/5 h (HT1376 pair) incubation in 2-NBDG-containing growth medium. ATP production (**B**) was assessed in cell lysates after cells reached confluence in 6-well plates. Mitochondrial activity (**C**) was assessed after 2 h incubation in MitoTracker-containing growth medium; MitoTracker Red and MitoTracker Green were used to measure mitochondrial polarization and mitochondrial mass, respectively. Mitochondrial activity was given by the mitochondrial polarization/mitochondrial mass ratio. * *p* < 0.05, ** *p* < 0.01, *** *p* < 0.005, **** *p* < 0.001, parental cells versus cisplatin-resistant cells.

The initial screening of the metabolic activities of the UBC cell lines (Figure 3) revealed a higher uptake of 2-NBDG (a glucose analogue) by HT1376R cells, in comparison to the parental cell line (Figure 3A). Increased extracellular glucose levels in HT1376R cells, indicative of glucose consumption, confirmed their glycolytic phenotype (Figure S3). The decreased ATP production (Figure 3B) aligned with reduced mitochondrial activity (Figure 3C), although the differences in this last parameter were not significant. Interestingly, the HT1376 pair presented the highest resistance ratio among all the cell lines studied, as previously mentioned (Figure 1B). Inversely, KU1919R cells presented an oxidative phenotype, characterized by significantly decreased 2-NBDG uptake (Figure 3A) and significantly increased mitochondrial activity (Figure 3C).

### 3.4. Antioxidant Activity

**Figure 4:**
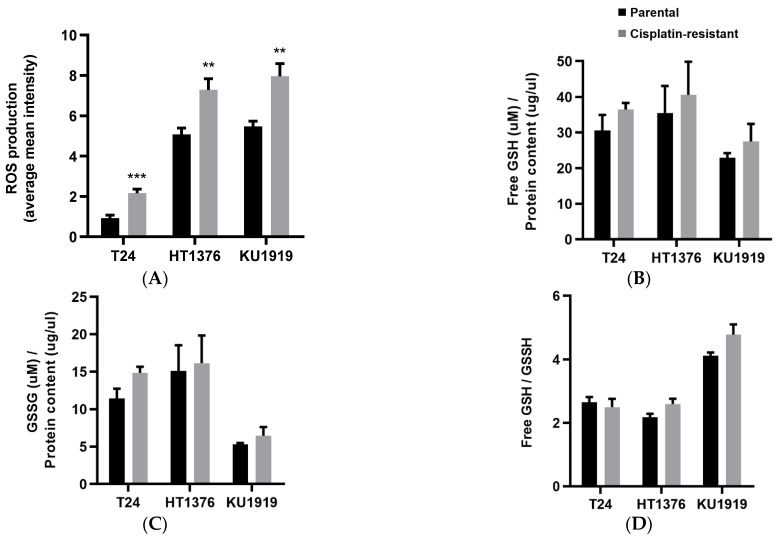
Antioxidant activity of isogenic pairs of urothelial bladder cancer cell lines. Reactive oxygen species (ROS, (**A**)) production was assessed after 30 min incubation in 2′,7′ –dichlorofluorescin diacetate (CM-H2DCFDA) in 1 × PBS. Free glutathione (GSH, (**B**)) and oxidized glutathione (GSSG, (**C**)) was assessed after cells reached confluence in T25 flasks. In (**D**), the free GSH versus GSSH ratio is shown. ** *p* < 0.01, *** *p* < 0.005, parental cells versus cisplatin-resistant cells.

Levels of ROS and glutathione were assessed in the isogenic pairs of UBC cell lines to evaluate their antioxidant activity (Figure 4). Regarding ROS production, significant differences were observed between the paired cell lines, with the highest levels being produced by the cisplatin-resistant sublines (Figure 4A). We did not detect any significant differences in free (GSH; Figure 4B) and oxidized (GSSG; Figure 4C) glutathione levels, as well as in their ratio (Figure 4D). Yet, a tendency for higher levels in HT1376R and KU1919R cells was evident, in comparison with their cisplatin-sensitive cell lines.

### 3.5. Lipid Metabolism

**Figure 5:**
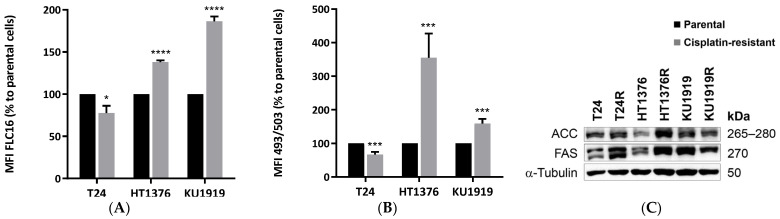
Lipid metabolism of isogenic pairs of urothelial bladder cancer cell lines. Palmitate uptake (**A**) and intracellular neutral lipid content (**B**) were detected after 30 min incubation with BODIPY™ FL C_16_ or BODIPY™ 493/503, respectively. * *p* < 0.05, *** *p* < 0.005, **** *p* < 0.001, parental cells versus cisplatin-resistant cells. Acetyl-CoA carboxylase (ACC) and fatty acid synthase (FAS) levels detected by Western blot ((**C**); results are representative of similar blots from three independent cell lysates; quantification of the results is shown in Figure S4).

To address lipid synthesis, we quantified Bodipy FL C16 uptake (Figure 5A) and BODIPY 493/503 (Figure 5B) by flow cytometry, as a measure of fatty acid uptake and intracellular neutral lipid content, respectively, in T24-, HT1376- and KU1919-paired UBC cells. T24R cells showed significantly lower levels of both components, while the inverse correlation was detected for HT1376R and KU1919R cells. We also detected significantly higher levels of acetyl-CoA carboxylase (ACC) and fatty acid synthase (FAS) in HT1376R cells (Figure 5C).

### 3.6. HT1376 versus HT1376R Cell Lines—Detailed Analysis of Metabolic Functions

**Figure 6:**
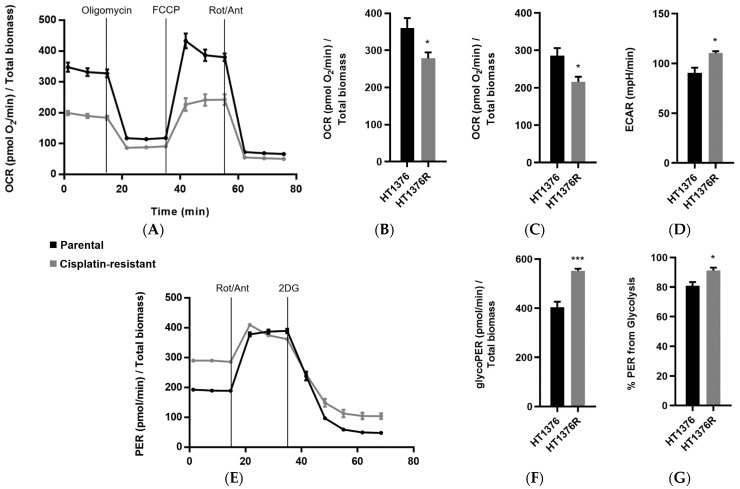
Cellular oxygen consumption rate (OCR) and extracellular acidification rate (ECAR) of an isogenic HT1376 pair of urothelial bladder cancer cell lines, measured in a Seahorse Analyzer using the Mito Stress Test and Glycolytic Rate Assay. The Mito Stress Assay profile (**A**), basal respiration (**B**), ATP production associated with oxygen consumption (**C**) and ECAR (**D**) were obtained through the Mito Stress Test. The Glycolytic Rate Assay profile (**E**), basal glycolysis (**F**) and proton efflux rate (PER) from glycolysis (**G**) were obtained through the Glycolytic Rate Assay. * *p* < 0.05, *** *p* < 0.005, parental cells versus cisplatin-resistant cells.

The HT1376 pair of cell lines revealed consistent differences regarding glycolytic metabolism and mitochondrial function. To confirm the previous results, the Mito Stress Test and a Glycolytic Rate Assay (Figure 6) were performed with these cells using a Seahorse Extracellular Flux Analyzer. Indeed, HT1376R cells showed an oxygen consumption rate (OCR) profile (Figure 6A) indicative of decreased basal respiration and oxygen consumption associated with ATP production (Figure 6B,C). In accordance, the extracellular acidification rate (ECAR) was also higher in the resistant subline (Figure 6D). The glycolytic rate assay profile (Figure 6E) showed that these cells presented higher basal glycolysis (Figure 6F,G), accompanied by an increase in proton efflux.

**Figure 7:**
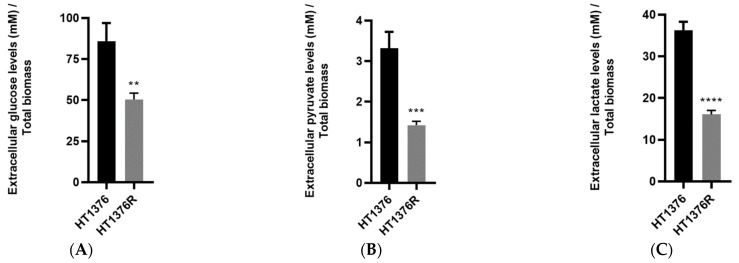
Extracellular glucose (**A**), pyruvate (**B**) and lactate (**C**) levels of the HT1376 isogenic pair of urothelial bladder cancer cell lines, measured by high-performance liquid chromatography. Metabolites were assessed in the growth medium 24 h post-incubation. ** *p* < 0.01, *** *p* < 0.005, **** *p* < 0.001, parental cells versus cisplatin-resistant cells.

Next, we used high-performance liquid chromatography (HPLC) to determine the glucose, pyruvate and lactate contents in the extracellular medium of the HT1376 paired cells (Figure 7). Glucose levels (Figure 7A) were significantly lower in the cisplatin-resistant cell line, confirming all of the above-mentioned results. Pyruvate levels (Figure 7B) were also significantly lower in HT1376R cells and, interestingly, lactate levels (Figure 7C) followed the same pattern.

**Figure 8:**
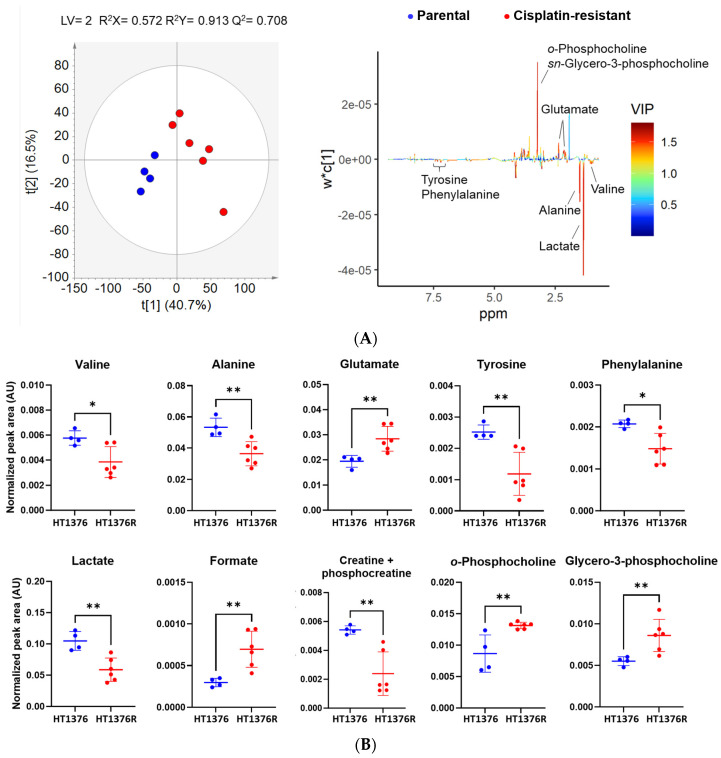

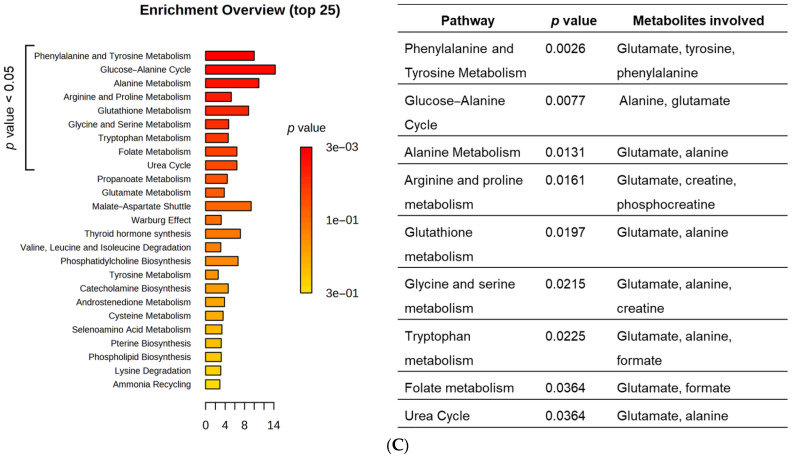
Intracellular metabolome analysis of the HT1376 isogenic pair of urothelial bladder cancer cell lines, determined by proton nuclear magnetic resonance (^1^H NMR) spectroscopy. Metabolites were assessed in the intracellular extracts 24 h post-incubation. The partial least squares discriminant analysis (PLS-DA) scores ((**A**), **left**) and loading plots (**A**), **right**) obtained for HT1376 (blue dots, *n* = 4) and HT1376R (red dots, *n* = 6) cells and graphical representation of the significantly altered metabolites (**B**); over-representation analysis (ORA, (**C**)) show significantly altered putative metabolic pathways. * *p* < 0.05, ** *p* < 0.01, parental cells versus cisplatin-resistant cells.

We further performed an intracellular metabolome analysis by ^1^H NMR-based metabolomics in the HT1376 pair, with unsupervised and supervised pairwise analyses among cisplatin-sensitive and resistant cells (Figure 8). A representative ^1^H NMR spectrum of the intracellular extract is shown in Figure S5. Multiple amino acids, organic acids, sugars and other components were assessed (in total, 22 metabolites). The PLS-DA model (Figure 8A) discriminated the cells with good predictive power (Q^2^ = 0.7). The intracellular content of the HT1376R cells revealed significantly lower levels of lactate, creatine plus phosphocreatine, valine, alanine, tyrosine and phenylalanine and significantly higher levels of glutamate, formate, *o*-phosphocholine and *sn*-glycero-3-phosphocholine (Figure 8B), when compared to their parental counterpart. Pathway analysis showed significant alterations in nine putative metabolic pathways (Figure 8C). Overall, resistance to cisplatin is associated with putative alterations in the metabolism of amino acids. The amino acid glutamate participates in most of those metabolic pathways.

## 4. Discussion

Cisplatin-based systemic chemotherapy is the standard of care in eligible advanced bladder cancer patients. The classical methotrexate, vinblastine, doxorubicin and cisplatin (MVAC) regimen or the less toxic gemcitabine and cisplatin (GC) scheme are the most used options [44], prolonging survival by about 14.8 and 13.8 months, respectively. However, half of these patients develop resistance [45]. By operating distinct yet not clearly identified signaling pathways, cisplatin resistance (CR) may be inherent or acquired during treatment [46]. By exposing different bladder cancer cell lines to escalating cisplatin doses in a long-term treatment (LTT), Skowron et al. found that LTT variants were not enriched in subpopulations with stem-like features that might have denoted inherent resistance, supporting that CR in UBC patients may occur under a post-treatment-acquired mechanism [47]. Similarly to the model used in Skowron’s study, we used three cisplatin-adapted UBC sublines in which acquired CR was developed following an intermittent stepwise selection protocol. All of the studied sublines presented a resistance profile two- to threefold higher than their parental counterparts. No apparent differences in cell growth kinetics were observed among the CR versus parental cells, which is in accordance with the results obtained by Vallo et al. [48] regarding T24 and HT1376 cells and precludes the influence of cell growth dynamics in the cisplatin response. Also, no differences were noted regarding the expression of basal levels of pro-apoptotic (cleaved PARP, caspase 9, Bim) and anti-apoptotic (Bcl-XL) markers.

With this study, we aimed to analyze the metabolic profile of UBC cells in the context of cisplatin resistance. For this, we used multiple approaches and methods encompassing an analysis of several metabolic pathways, in order to cross-validate our results and gain a comprehensive understanding of the metabolic alterations occurring in cisplatin-sensitive and resistant cancer cells. We observed the most striking and consistent differences between parental and resistant cells in the HT1376 pair, for which we found the highest resistant ratio. The HT1376 cell line, originating from a grade 3 carcinoma of the urinary bladder, presents a near-tetraploid karyotype, depicting TP53, RB1 and PTEN losses (yet no alteration of the PIK3CA gene region) [49], which is compatible with an invasive and metastatic profile [50]. It represents, therefore, a suitable model of advanced disease. HT1376 drug-adapted cells showed increased levels of GLUT3, an increased demand for glucose, higher basal glycolysis and decreased basal respiration. Decreased ATP production and mitochondrial activity were concordant with decreased basal respiration and oxygen consumption associated with ATP production. Seahorse analysis also revealed an increase in the proton efflux and extracellular acidification rate. However, while intracellular lactate levels were lower, extracellular lactate levels were also lower in the HT1376R cell culture medium. These apparently conflicting results are much likely related to the temporal window of the assays. In fact, all of the functional analyses were performed at 24 h post-incubation (2-NBDG uptake was evaluated after 16 h of glucose starvation), with the exception of the Seahorse technology, which performs a real-time cell metabolic analysis. Thus, the Seahorse results support the occurrence of an exacerbated glycolytic phenotype in the CR cells. HT1376R cells were also used by our group in previous works, in which a re-sensitization to cisplatin was observed upon depletion of glucose uptake or lactate transport through pharmacological treatment with the HK2 inhibitor 2-deoxy-D-glucose [24] or with the MCT1 specific inhibitor AZD3965 [51], respectively. These results corroborate previous findings of the preponderance of the glycolytic metabolism in a CR setting in bladder cancer [23,26,30,33]. This phenotype has also been observed in other malignancies, namely osteosarcoma [52] and ovarian [53,54], lung [55] and gastric cancers [56], in which several effectors of the glycolytic phenotype such as HK2 [54], enolase [56], PKM2 [52], MCT1 [53] and CD147 [57] were overexpressed and correlated with cisplatin resistance, while their genetic or pharmacological inhibition restored chemosensitivity.

Curiously, HT1376R cells had low levels of MCT4, a monocarboxylate transporter that preferentially exports lactate from highly glycolytic cells. However, this function is redundant with MCT1 functions (adapted for both the uptake and efflux of monocarboxylate from cells) [57], whose levels were comparable among the parental and resistant cells. Moreover, protein levels of CD147 were detected in the HT1376 pair, co-localizing with both MCTs, as expected by its obligate chaperoning function [58]. Theoretically, HT1376R cells should be able to compensate for MCT4 absence with MCT1 presence and, therefore, lactate exportation is assured. Of note, in a previous work where we used these low-MCT4-expressing HT1376R cells together with another cell line showing higher levels of the MCT4 (253J cell line, anticancer activity and re-sensitization to cisplatin upon MCT1 inhibition was largely dependent on the lack of MCT4 expression [51], which corroborated the interchangeable roles of the transporters [59] and underpinned the importance of MCT1 on lactate exportation in HT1376R cells.

All of the studied cell lines were consuming glucose—to a higher extent in the resistant sublines, mainly in HT1376R cells—which indicates the presence of a functional transporter. Decreased levels of GLUT1 in HT1376R cells were probably compensated by GLUT3, for which a higher expression was obtained in this CR subline. In the study by Li et al., GLUT1 mediated CR in parental UBC cells, as its targeting by miR-218 enhanced cisplatin sensitivity by decreasing glucose uptake, while GLUT1 upregulation restored chemoresistance [33]. GLUT1 overexpression has also been associated with CR in esophageal [60], laryngeal [61], oral [62] and gastric cancers [63]. However, GLUT3, the major neuronal glucose transporter, has also been shown to be upregulated in malignancy [64,65,66] apart from brain tumors [67], including in bladder cancer [68], and it was surely operating in our UBC cell models in the present study. GLUT3 overexpression in glioblastoma cell lines was associated with increased glycolysis and therapeutic resistance [69,70].

As previously mentioned, the lower lactate levels observed in the extracellular medium of HT1376R cells, when compared to the parental subline, are intriguing. On the one hand, the real-time cell metabolism results are compatible with an exacerbated glycolytic phenotype, and we also observed that 24 h after cells’ incubation glucose uptake was still higher in these cells and ATP production was still lower. On the other hand, at this timepoint, extracellular lactate levels were lower, although they were also lower intracellularly. This last aspect was expected, as clearance of excess lactate production due to enhanced glycolysis must occur to assure an alkaline intracellular content and cancer cells’ viability. Lower extracellular lactate levels probably indicate that lactate was being rapidly uptaken by the cells and used to replenish other metabolic pathways. In fact, this metabolic symbiosis was proposed by our group in a clinical study, where we described that catabolic glycolytic cancer and stromal cells should be metabolically coupled to anabolic oxidative cancer cells in a “reverse Warburg effect” [71] fashion and that this phenotype is related with cisplatin resistance [27]. While the present results probably indicate the occurrence of a lactate shuttle, they are not sufficient to sustain that a metabolic shift occurred in the CR cells, as demonstrated by others (reviewed in [72]), and that both glycolytic and oxidative cancer cells were co-existing in the culture. Moreover, the fact that we were only working with cancer cells in two-dimensional cultures constitutes a limitation; co-culture systems with cancer and stromal cells could have provided us with important information regarding metabolic heterogeneity in the UBC setting. However, we cannot discard this hypothesis, as although the real-time analysis clearly indicated mitochondrial dormancy in the HT1376R cells, at 24 h post-cell incubation, no significant differences were observed between the paired cells, and they were indeed exhibiting mitochondrial activity. In the study by Duan et al., glycolysis-dependent glioma cells switched to an oxidative metabolism upon glucose-limiting conditions, with this metabolic reprogramming being induced by lactic acid [73]. Of note, in our study, the spent culture medium of HT1376R cells at 24 h post-incubation had the lower glucose levels of all cultured cell lines, which possibly explains their need to uptake lactate. Cancer cells are known by their flexible and plastic metabolisms, which means they can use other metabolic substrates besides glucose (the main energy source) and rewire metabolic pathways when facing a glucose-deprived microenvironment [74]. Ying et al. [75] experimentally induced glucose starvation in breast, cervical and lung cancer cells and observed that not only lactate but also glutamine (an alternate energy source that will be discussed later) were used to produce NADPH, an important cofactor that provides the reducing potential for anabolic reactions and redox balance [76], through distinct metabolic pathways. In our study, HT1376R cells probably adapted their metabolism in response to the increasing lack of glucose and to the immediate availability of substitute substrates.

While the glycolytic phenotype seems to assume a determinant role in cisplatin resistance in HT1376R cells, additional mechanisms beyond the Warburg and the reverse Warburg effect are likely playing a role here. When compared to their parental counterparts, HT1376R cells presented a significantly enhanced lipid metabolism, as seen by the higher neutral lipid content and palmitate uptake, increased levels of enzymes involved in fatty acid synthesis, and increased levels of short-chain fatty acids and intracellular intermediates for phospholipid synthesis. Lipid metabolism is among the most important metabolic adaptations in cancer, contributing to cancer cell survival and growth and tumor progression [77]. Yang et al. recently identified and validated a 6-lipid metabolism-related gene signature for the prognosis prediction of UBC patients [78]. Lipid metabolism is also closely related to cisplatin resistance. In accordance with our results, in UBC T24 CR cells, enzymes and precursors involved in fatty acid synthesis were increased, and targeted inhibition of this process reduced chemoresistance [79]. Similar results were seen in other malignancies [80,81,82]. In the study by Tan et al. [83], a shift from glucose towards lipid energy metabolism in CR ovarian cancer cells was demonstrated by diminished glucose uptake and augmented fatty acid uptake and lipogenesis. Interestingly, Ippolito et al. [84] described a metabolic–epigenetic regulatory mechanism involving histone acetylation, in which the expression of lipid metabolism-related genes was increased by cancer-associated fibroblast-secreted lactate. On the other hand, lactate promotes glutamine uptake, and the use of glutamine and lactate for acetyl-CoA synthesis is an additional route for lipid biogenesis and metabolism regulation [85]. Pérez-Escuredo et al. demonstrated that intracellular lactate signaling promoted the uptake of glutamine and its subsequent metabolism in oxidative malignant cells [86]. It is irrefutable that glutamine uptake also affects chemotherapy sensitivity, as shown by higher levels of SLC1A5 (glutamine transporter) in CR lung cancer cells when compared to wild-type cells [87] and chemoresistance induction upon SLC1A5 overexpression in pancreatic cancer cells [88]. In our study, we did not evaluate glutamine uptake, but significantly higher levels of glutamate, the immediate product of glutamine metabolism produced by glutaminase action [89], were detected in HT1376R cells. Cancer cells are addicted to glutamine, as it provides both nitrogen for the biosynthesis of nucleotides and amino acids and carbon for lipogenesis and Krebs cycle replenishment. Glutamine also controls GSH homeostasis, elevating its intracellular levels through NADPH/NADP^+^ ratio maintenance and preventing GSSG release from the cells and its extracellular degradation [89]. In our study, a tendency for higher levels of free GSH and a higher GSH/GSSG ratio in HT1376R cells was noted, which is in accordance with the role of this important component of the cellular antioxidant system in chemoresistance. In fact, elevated GSH levels and activation of GSH-related enzymes are involved in resistance to therapy [90,91], and drugs targeting GSH biosynthesis were able to re-sensitize neuroblastoma cells [92]. On the other hand, GSH degradation by γ-glutamyl transferase releases cysteine, glycine and glutamate [93]. It is possible that some of the glutamate seen at higher levels in our CR cells resulted from GSH degradation.

Significant differences regarding basal ROS levels were observed in the paired cell lines in our study, with higher ROS levels being produced by all cisplatin-resistant UBC cell lines. Accumulation of ROS is one of the molecular mechanisms that lies behind the cytotoxic properties of cisplatin [94]. In accordance with our results, Shirato et al. observed that basal levels of intracellular ROS were significantly lower in CR HT1376 cells, when compared to the parental subline; accordingly, cisplatin exposure increased ROS levels in the parental cells and had no effect on the resistant cells [95].

HT1376R cells revealed a metabolic profile clearly compatible with an exacerbation of the glycolytic metabolism, as well as the adoption of other metabolic routes upon lactate dependence, corroborating that the reprogramming of glycometabolism, lipid metabolism, amino acid metabolism and other energy metabolism pathways are indeed involved in cisplatin resistance [96]. Yet, differences among the remaining pairs probably reveal distinct scenarios. Regarding the KU1919 pair, a lower glucose uptake and a concomitant higher mitochondrial activity in KU1919R cells presumably indicates a preference for oxidative phosphorylation over aerobic glycolysis. Curiously, this metabolic rewiring, with cisplatin-resistant cells recovering their preference for OXPHOS, has been described by some authors [97,98,99,100], in which reliance on OXPHOS was concomitant with increased oxygen consumption and overexpression of Bcl-2 [99] and increased ROS production and glutamine uptake [98]. Nevertheless, Dar et al. demonstrated that CR ovarian cancer cell lines were able to reprogram their metabolism from OXPHOS to glycolysis, and vice versa, highlighting this heterogeneity as an important survival mechanism [101]. Another interesting result of our work was the fact that KU1919R cells also showed, similarly to HT1376R cells, increased fatty acid uptake and intracellular neutral lipid content, in comparison to KU1919 cells, which underpins an accelerated lipid metabolism. Curiously, fatty acid uptake was higher in KU1919R cells, while neutral lipid content was higher in HT1376R cells, pointing to increased fatty acid β-oxidation in the HT1376R cell line. Therefore, the link between cisplatin resistance and a specific metabolic pathway is not straightforward. Notwithstanding, metabolic reprogramming seems to occur indeed, and it is probably dependent on the tissue of origin of the cells, as well as their variable genetic backgrounds.

## 5. Conclusions

In this work, we studied the metabolic profiles of three pairs of cisplatin-sensitive (CS) and cisplatin-resistant (CR) UBC cell lines. Two of the CR cell lines exhibited an increased reliance on glycolytic metabolism, particularly evident for HT1376 CR cells, which showed a higher rate of cisplatin resistance. However, within this specific cell line, there was a transition towards alternative metabolic pathways over time, including the increased uptake of lactate, synthesis of lipids and alterations in glutamate metabolism. Conversely, the other cell line appeared to undergo metabolic reprogramming, reverting back to a preference for oxygen consumption. Thus, it is evident that cisplatin resistance in bladder cancer involves profound alterations in several metabolic pathways beyond the Warburg effect, which are likely dependent on the molecular signature of each cell line, with phenotypes encompassing not only an increased glycolytic metabolism, but also lactate uptake, increased lipid biosynthesis, amino acid metabolism and re-reprogramming towards an oxidative phenotype. Further studies, namely a deep gene expression profiling in suitable preclinical models, in parallel with the investigation of the mechanistic insights that sustain the aforementioned alterations, are needed to unravel possible nuances that surely lie behind the unique genotype of cisplatin-resistant bladder cancer cells. Those studies will certainly facilitate the advent of personalized cisplatin resistance reversal strategies based on the tumor metabolic signature of UBC patients.

## Data Availability

All of the data generated during this study are included in the main article and in the Supplementary Materials. Further enquiries can be directed to the corresponding author.

## Supplementary Materials

The following supporting information can be downloaded at: https://www.mdpi.com/article/10.3390/cancers16071418/s1, Table S1: The antibodies used for Western blotting and immunofluorescence; Figure S1. The representative Western blots of baseline levels of cell death biomarkers in isogenic pairs of urothelial bladder cancer cell lines (A), their respective quantification (B) and the original Western blots (C); Figure S2. The quantification of the Western blot results shown in Figure 2A (A) and the original Western blots (B); Figure S3. The glucose consumption of isogenic pairs of urothelial bladder cancer cell lines. Figure S4. The quantification of the Western blot results shown in Figure 5 (A) and the original Western blots (B); Figure S5. The typical ^1^H NMR spectra of the intracellular extract.
