# Supplementary material for: Cisplatin-Resistant Urothelial Bladder Cancer Cells Undergo Metabolic Reprogramming beyond the Warburg Effect
Source: Cancers (Basel). 2024 Apr 5;16(7):1418. doi: 10.3390/cancers16071418 (PMC11010907; doi:10.3390/cancers16071418)
Supplement: Supplementary file 1 [file cancers-16-01418-s001.zip › cancers-2939840-supplementary.pdf]

# - SUPPLEMENTARY MATERIALS -

Table S1. Antibodies used for Western blotting (WB) and immunofluorescence (IFC).

	Antibody	Reference (Company)	WB dilution	IFC dilution
Primary antibodies	**Bcl-xL**	sc-8392 (Santa Cruz Biotechnology®)	1:300	-
	**ACC**	#3662 (Cell Signalling® Technology)	1:1000	
	**Bim**	#2933 (Cell Signalling® Technology)	1:1000	-
	**Caspase 9**	#9508 (Cell Signalling® Technology)	1:500	-
	**CD147**	sc-71038 (Santa Cruz Biotechnology®)	1:500	1:500
	**FAS**	sc-48357 (Santa Cruz Biotechnology®)	1:2000	
	**GLUT1**	ab15309 (AbCam)	1:500	-
	**HIF1α**	#610958 (BD Biosciences)	1:500	
	**HK2**	ab104836 (AbCam)	1:2000	
	**LDHA**	sc-100775 (Santa Cruz Biotechnology®)	1:1000	-
	**MCT1**	sc-365501 (Santa Cruz Biotechnology®)	1:500	-
		AB3538P (Chemicon®)	-	1:200
	**MCT4**	sc-50329 (Santa Cruz Biotechnology®)	1:500	1:500
	**PARP**	#9542 (Cell Signalling® Technology)	1:500	-
	**PDK**	sc-28783 (Santa Cruz Biotechnology®)	1:500	
Secondary antibodies	**m-IgGκ BP-HRP**	sc-516102 (Santa Cruz Biotechnology®)	1:2500	
	**IgG-HRP**	sc-2357 (Santa Cruz Biotechnology®)	1:2500	
	**Alexa Fluor® 594**	A11032 (Invitrogen™)		1:500
	**Alexa Fluor® 488**	A11008 (Invitrogen™)		1:500
Loading controls	**β-Actin**	sc-8432 (Santa Cruz Biotechnology®)	1:500	
	**α-Tubulin**	ab15246 (AbCam)	1:2500	

A

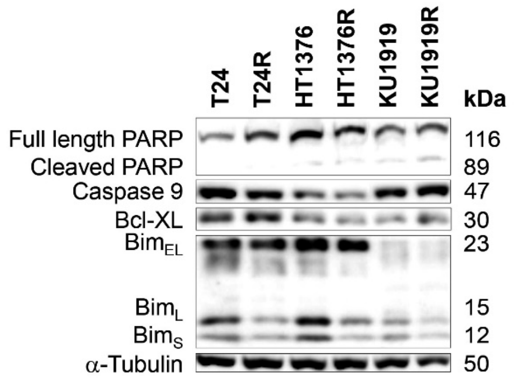

B

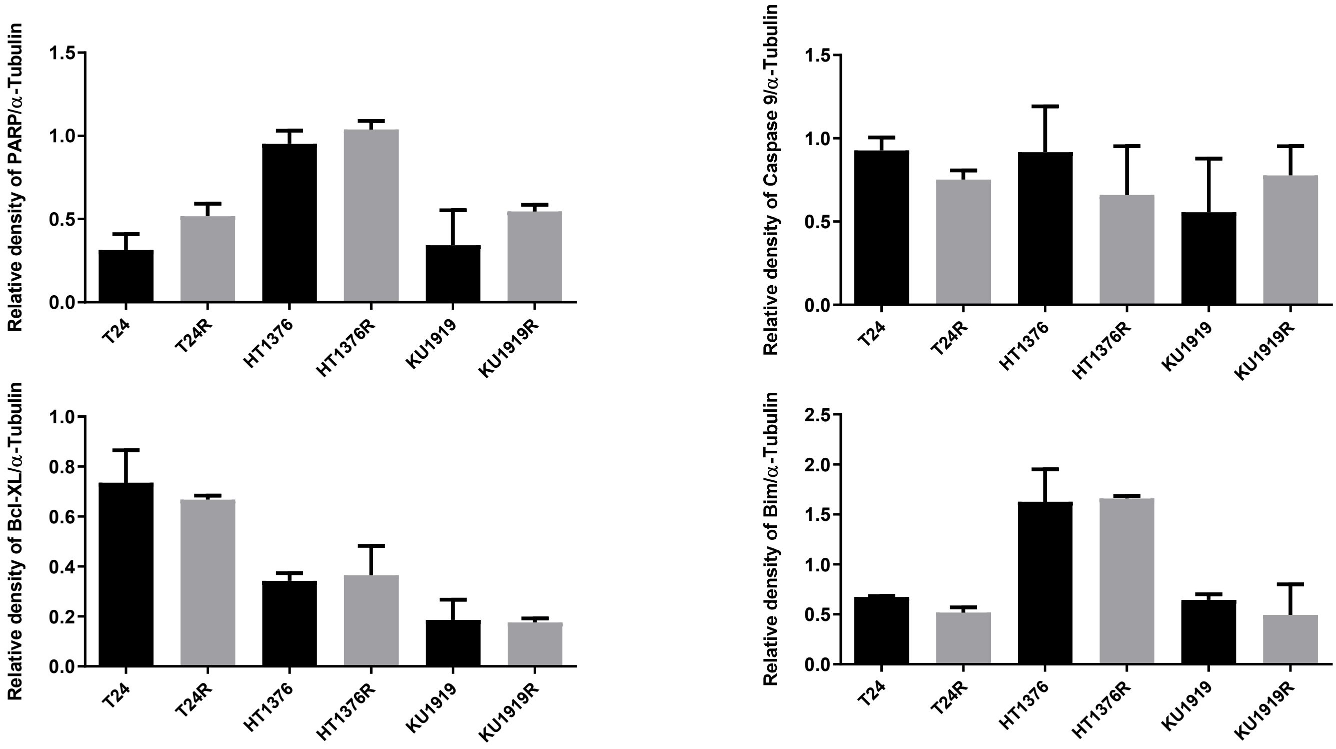

C

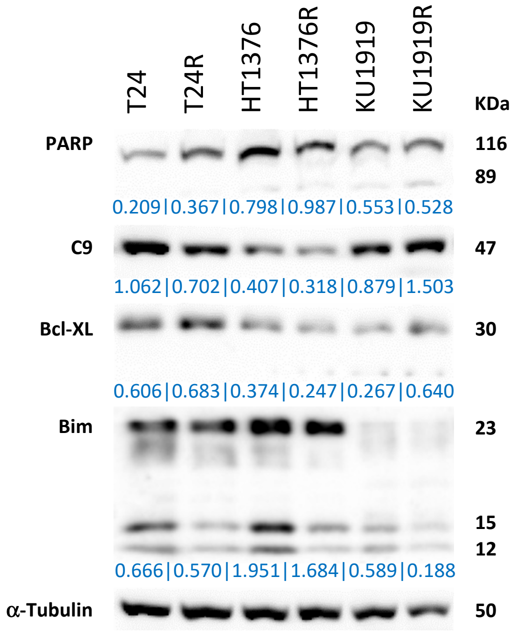

Figure S1. The representative Western blots of baseline levels of cell-death biomarkers in isogenic pairs of urothelial bladder cancer cell lines (A), their respective quantification (B) and the original Western blots (C). Results are representative of similar blots from three independent cell lysates. Intensity ratios relative to α-Tubulin are shown in blue.

A

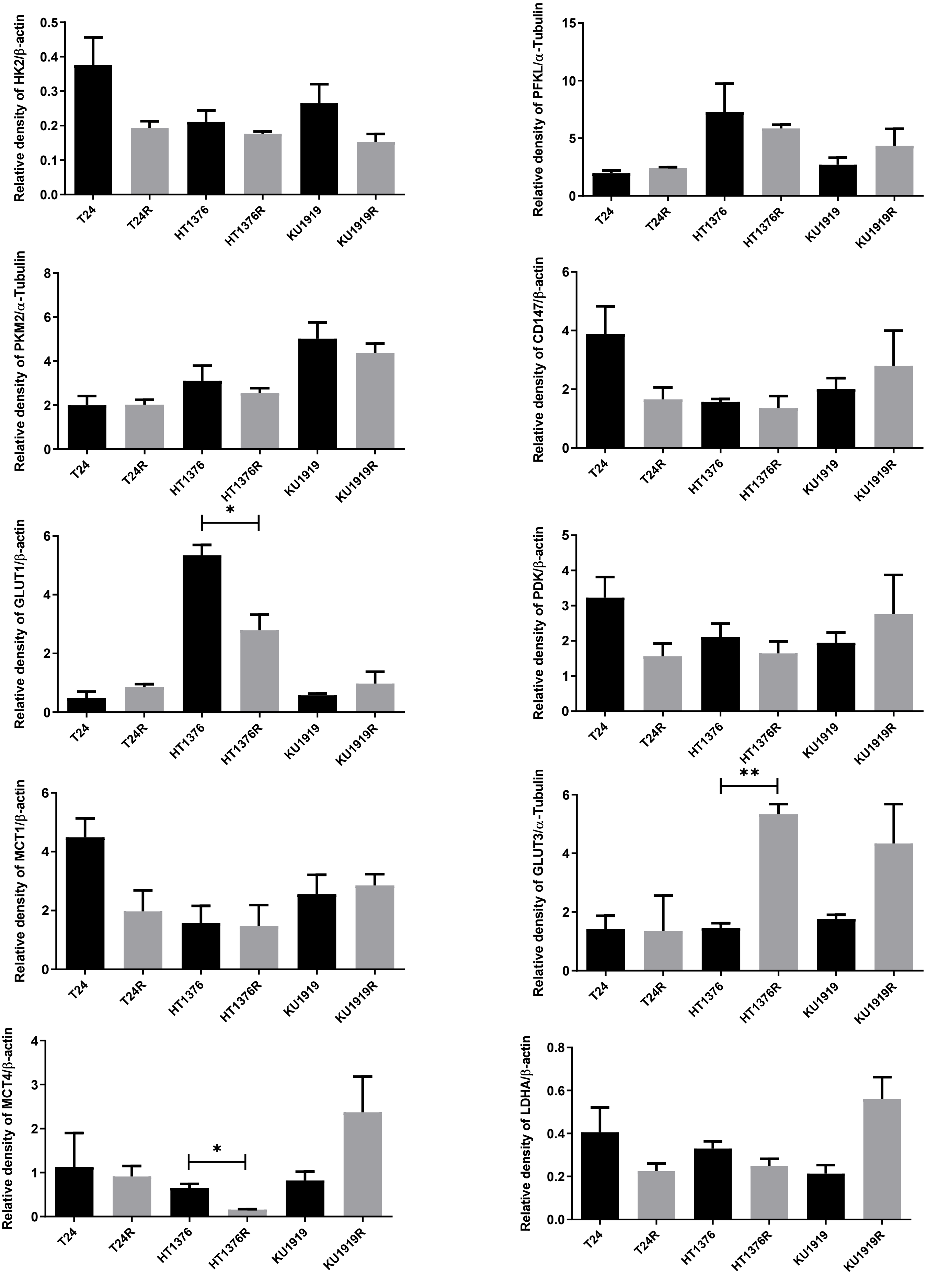
Relative density of HK2/β-actin
0.0
0.1
0.2
0.3
0.4
0.5
Relative density of PFKL/α-Tubulin
0
5
10
15
Relative density of PKM2/α-Tubulin
0
2
4
6
8
Relative density of CD147/β-actin
0
2
4
6
Relative density of GLUT1/β-actin
*
0
2
4
6
Relative density of PDK/β-actin
0
1
2
3
4
5
Relative density of MCT1/β-actin
0
2
4
6
Relative density of GLUT3/α-Tubulin
**
0
2
4
6
Relative density of MCT4/β-actin
*
0
1
2
3
4
Relative density of LDHA/β-actin
0.0
0.2
0.4
0.6
0.8
T24
T24R
HT1376
HT1376R
KU1919
KU1919R

B

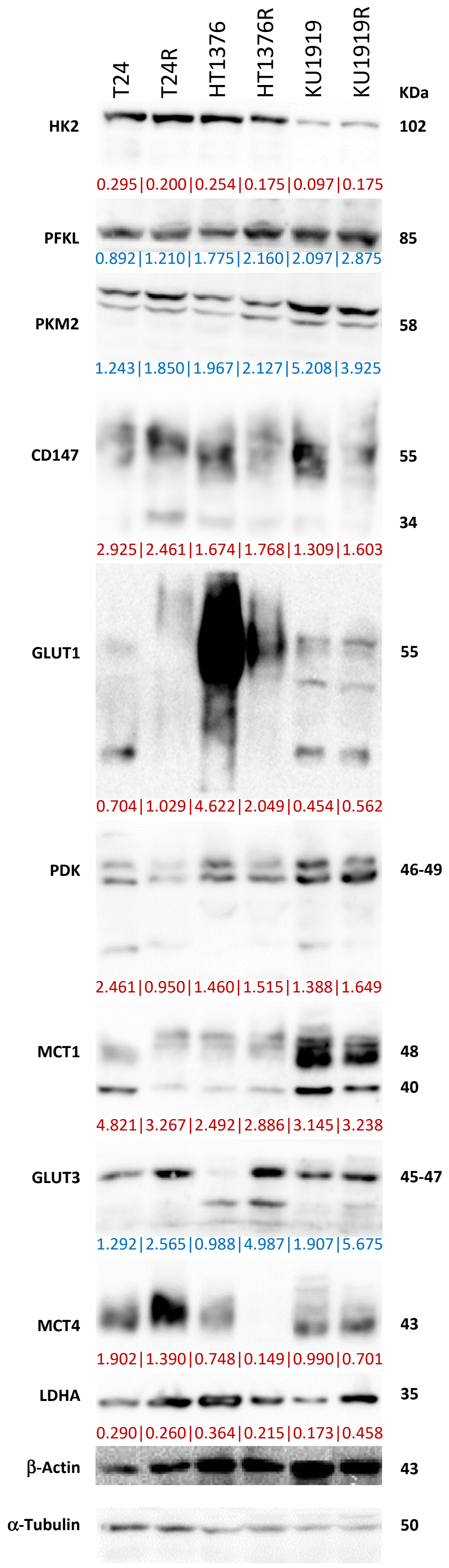

Figure S2. Quantification of the Western blot results shown in Figure 2A (A) and the original Western blots (B). Intensity ratios relative to β-Actin and α-Tubulin are shown in red and in blue, respectively. *$p < 0.05$, parental cells *versus* cisplatin-resistant cells.

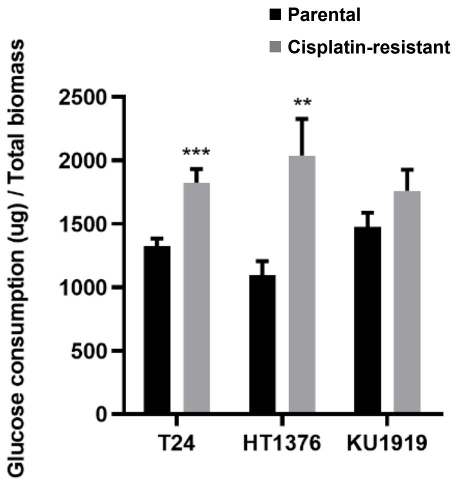

Figure S3. Glucose consumption of isogenic pairs of urothelial bladder cancer cell lines, assessed in the growth medium 24h post-incubation. ** $p < 0.01$, ***$p < 0.005$, parental cells *versus* cisplatin-resistant cells.

A

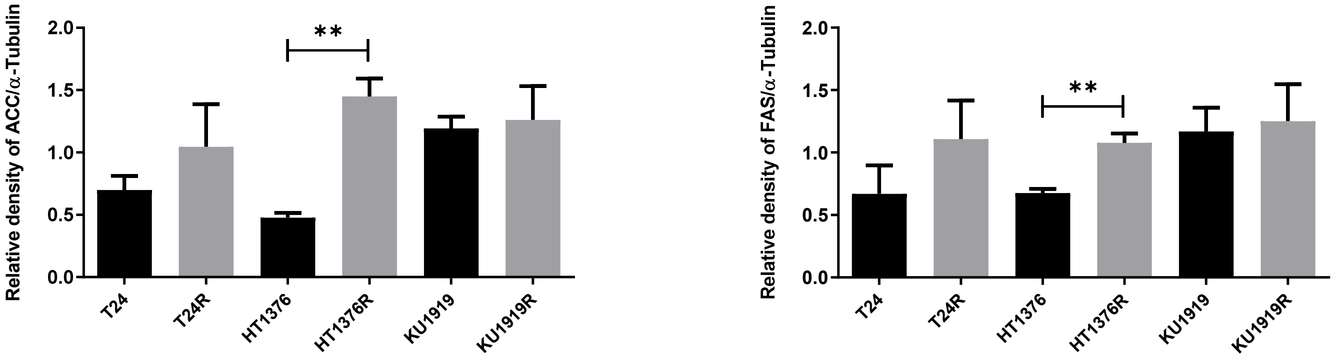

B

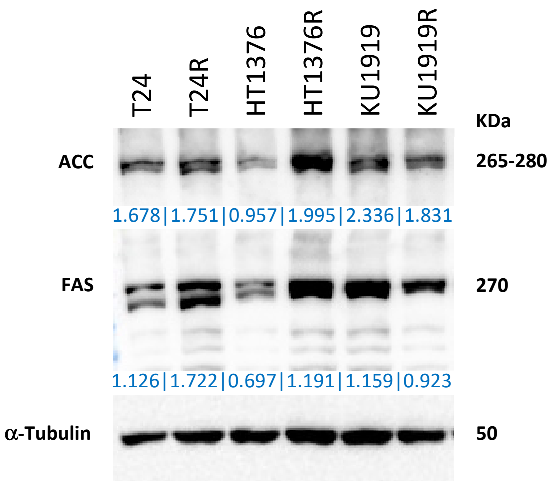

Figure S4. Quantification of the Western blot results shown in Figure 5 (A) and the original Western blots (B). Intensity ratios relative to α-Tubulin are shown in blue. **$p < 0.01$, parental cells *versus* cisplatin-resistant cells.

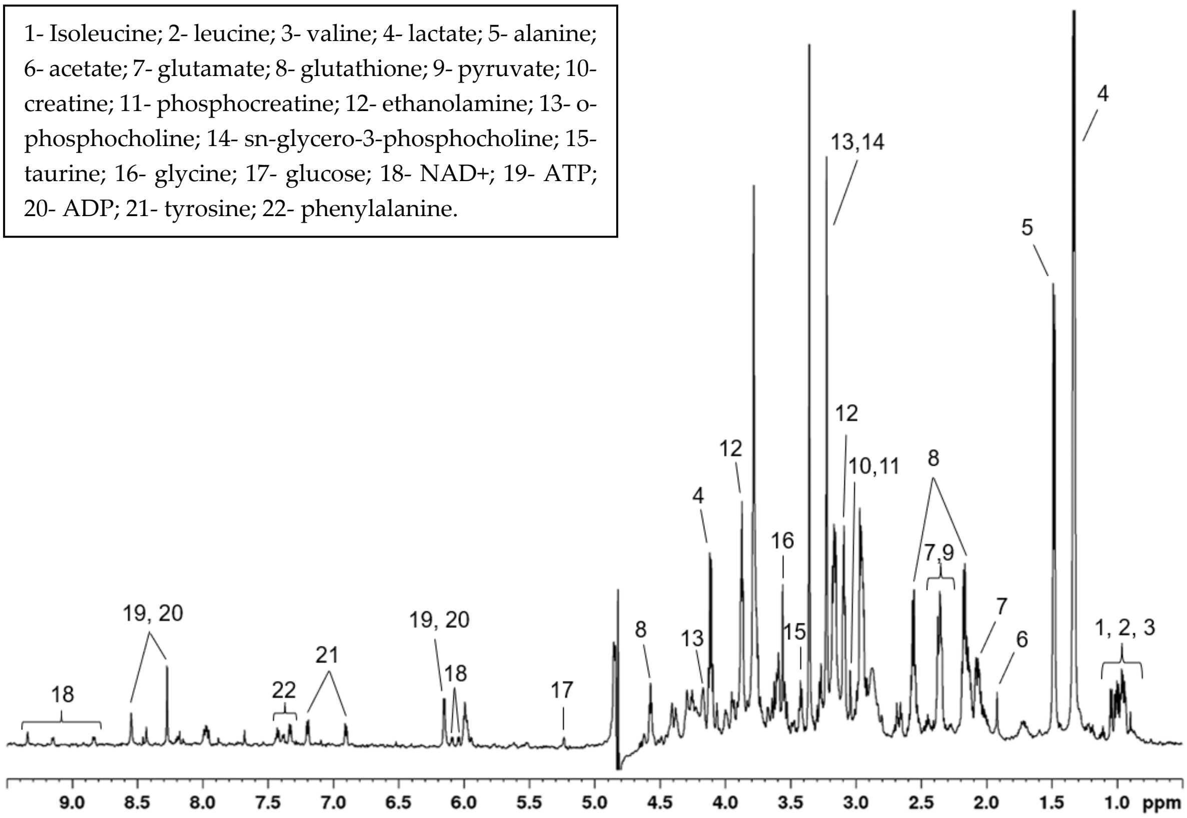

Figure S5. Typical ^{1}H NMR spectra of the intracellular extract.
